# Early Detection of Cognitive Decline Using an Evidence‐Based Risk Score and Subjective Memory Concerns in an Ethnoracially Diverse Sample

**DOI:** 10.1002/alz70860_104660

**Published:** 2025-12-23

**Authors:** Alexa S. Gonzalez, Andrea P. Ochoa Lopez, Stephanie Torres, Luis D. Medina

**Affiliations:** ^1^ University of Houston, Houston, TX, USA; ^2^ Perspectives Psychological, Newport Beach, CA, USA

## Abstract

**Background:**

Dementia risk scores are calculated from various health risk factors to predict one's chances of developing dementia in the future, contributing to early identification of Alzheimer's disease and related dementias (ADRDs). However, prior research suggests risk scores not accounting for race/ethnicity are less predictive of cognitive performance in diverse samples. We evaluated the Mexican American Dementia Nomogram (MADeN) score and its association with subjective and objective cognition in a sample of Hispanic/Latin American (H/L), non‐Hispanic Black, and non‐Hispanic White individuals.

**Method:**

Data from 3,634 participants (Age=65±8.65; Education = 13.4±4.23) in the Health and Aging Brain Study: Health Disparities were examined (H/L: 1,348; Black: 1,002; White: 1,284). Modified MADeN scores were calculated based on female gender, age >65, <4 years of education, low social support, limited community involvement, diabetes, depression, impaired instrumental activities of daily living, and limited mobility. Nested regression models examined relationships between MADeN scores, self‐reported subjective memory concerns (SMCs), and a cognitive performance composite derived from a comprehensive battery. Models were tested across ethnoracial groups and stratified by race/ethnicity.

**Result:**

In the full sample, modified MADeN scores accounted for 15.1% of variance in SMC endorsements (*β* =0.40, *p* <0.001, 95% CI [0.37, 0.43]) and 9.2% of variance in cognitive performance (*β* = ‐0.10, *p* <0.001, 95% CI [‐0.11, ‐0.09]). Stratified models suggested MADeN scores explained similar variance in SMCs across all three groups (range=13.6% to 15.4%; all *p*s<0.001). MADeN scores explained 13.3% of variance in cognitive performance for H/Ls, 5.2% for Black individuals, and 2.3% for White individuals (all *p*s<0.001). SMCs significantly explained additional variance in cognition for Black (Δ*R* ^2^=0.026) and White individuals (Δ*R* ^2^=0.09), but not for H/Ls (*p* = 0.066).

**Conclusion:**

Individuals with higher MADeN scores are likely to endorse more SMCs and have poorer cognitive performance. MADeN scores appear most effective for detecting risk in H/Ls and are moderately meaningful for Black individuals, while SMCs are more informative for non‐Hispanic White individuals. Future research may entail examining potential moderators between MADeN scores and cognition, like ADRD biomarkers.

